# The Understanding of Human Death by Polish Early Career Pre-Specialist Physicians

**DOI:** 10.3390/ijerph192416573

**Published:** 2022-12-09

**Authors:** Krzysztof Leśniewski, Bożena Baczewska, Beata Antoszewska

**Affiliations:** 1 Department of Orthodox Theology, Faculty of Theology, The John Paul II Catholic University of Lublin, Al. Racławickie 14, 20-950 Lublin, Poland; 2 Department of Internal Medicine and Internal Medicine in Nursing, Faculty of Health Sciences, Medical University of Lublin, Chodźki 7, 20-093 Lublin, Poland; 3 Department of Special Needs Pedagogy and Resocialization, Faculty of Social Sciences, The University of Warmia and Mazury in Olsztyn, Żołnierska 14, 10-561 Olsztyn, Poland

**Keywords:** death, brain death, neurological criteria of death, attitudes toward brain death

## Abstract

Despite the legal classification of cerebral death as the actual death of a human being and the continuous clarification of neurological criteria, the subject of death, particularly, when exactly it occurs, has been the subject of debate not only in the medical environment but also in other scientific communities for over sixty years. This issue is also present in social discourse. In Poland, as well as in other countries, the concepts of “death” and “cerebral death” have a legal basis. Considerations devoted to death are also important for tanatopedagogics, which focuses primarily on mortality. Indeed, the quality of relationships with other people depends to a large extent on the awareness of death. The study involved 113 pre-specialist physicians employed in various medical centers in Poland. An original questionnaire was used to study the understanding of human death in the light of legal and medical acts that came into force between 2007 and 2019. The study showed that only 7.08% of pre-specialist physicians could fully and correctly identify the basis for declaring a patient dead after diagnosing the irreversible cessation of brain function, and only 33.63% of all respondents understood death in accordance with legal acts currently in force in Poland. Moreover, nearly half of the study participants (47.79%) indicated that irreversible loss of consciousness is not adequate grounds for determining a patient’s death, while 56.64% felt that cerebral death is equal to the biological death of a human being. Women were significantly more likely to understand the concept of death (*p* = 0.028) as defined by current documents and to perceive the irreversible loss of consciousness as an insufficient basis for determining a patient’s death (*p* = 0.040) and also to correctly indicate on what basis cerebral death is identified with human death (*p* = 0.003), as expressed by current legal regulations in Poland.

## 1. Introduction

Although the moment of human death is a mystery [1], traditionally, death has been believed to occur when breathing and circulation cease. Accordingly, “physical death” (Latin *mors, exitus letalis*) is a state characterized by the loss of vital signs. In order to determine physical death, it is necessary to notice the irreversible loss of cardiac and respiratory function. Herein, clinical tests are helpful in order to determine whether the circulatory–respiratory criterion is met. For centuries, the permanent cessation of vital functions has been used by physicians to declare death [2]. However, physical criteria used to determine death are not intended to indicate its exact moment but rather to identify the point in time that it has already happened [3]. According to Aristotle, a human being lives as long as he or she possesses an inner unity of matter and form (Physics I.7; De Anima II.2) [4]. In practice, this implies that the unity of a living organism is formed by an anti-entropic process of integration. From this perspective, death is the end of the integration of the human organism and the beginning of the decomposition of its body [5].

From the end of the 1960s, human death also began to be declared based on neurological criteria relating to the functioning of the brain [6]. It was at that time that the exact moment of death had to be identified, by mutual agreement between medical specialists, as a specific event in time [7]. The reason why the moment of death had to be indicated very precisely and quickly was to allow organs to be harvested from a donor in the best possible condition [8]. The diagnosis of death based on the cessation of brain function was a revolutionary change compared to the so-called “classical definition”, which is based on cardiopulmonary criteria [9]. Until the introduction of the neurological criteria of death, the decomposition of the body due to the entropic process was considered to be the only reliable sign of death [10]. The issue of the end of human biological life demands a distinction between the concept of death and the criterion, or set of criteria, on the basis of which it is established. The concept or definition of human death belongs to the philosophical and theological sphere, as it requires reflection on ideas such as the loss of the integrative unity of the organism, the loss of personhood, arbitrary stipulation by society and the separation of the soul from the body. In turn, the criterion of death is a fact relating to the sphere of physiology, which is a sign of death defined at the level of an accepted anthropological concept. Each criterion of death is based on a certain concept and assumes specific judgments about its physical signs in a manner indicating that death, as defined by such judgments, has actually occurred [5].

It is worth noting that despite the legal classification of cerebral death as the actual death of a human being and the continuous clarification of neurological criteria, the moment of death has been an ongoing aporia in many medical circles for over sixty years [11]. In the scientific debate on the understanding of death and cerebral death, as well as the criteria for its determination, specialist physicians, bioethicists [12,13,14,15], lawyers [16,17], philosophers [18,19], theologians [20] and pedagogues [21,22,23] present various bio-physiological, philosophical, social and theological arguments.

In Poland, as well as in other countries, the concepts of “death” and “cerebral death” have a legal basis. Between 2007 and 2019, the basic legal act in this respect was the Notice of the Minister of Health of 17 July 2007 on the Criteria and Method of Determining the Permanent Irreversible Cessation of the Brain Function, based on Art. 9 sec. 3 of the “Cell, Tissue and Organ Recovery, Storage and Transplantation Act of 1 July 2005” (acronym NMH2007) (Dz. U./Journal of Laws/No. 169 item 1411). As of 2019, the Notice of the Minister of Health of 4 December 2019 on the Manner and Criteria for Determining Permanent Irreversible Cessation of Brain Function, having its legal basis in Art. 43a sec. 3 point 1 of the “Act of 5 December 1996 on the Professions of Doctor and Dentist” (acronym NMH2019) (Dz. U./Journal of Laws/of 2019, item 537, 577, 730 and 1590), is in force. Table 1 below shows the differences in defining human death and the essential problems in determining it in Polish legal and medical documents from 2007 and 2019.

In this reported study, the Interdisciplinary Research Team undertook to conduct research on the understanding of human death by pre-specialist physicians due to the global medical and legal discussion regarding the understanding of human death. The intention of the researchers was not to express their views on the global debate on the concept of human death, nor to evaluate the views of the surveyed doctors, but to analyze the statements they chose regarding death and its confirmation in the light of the law in force in Poland at that time. During our research, in Poland, the legal and medical regulation, NMH2007, was in force. It stated that “death is a dissociated phenomenon”, that is, according to the Legislator, death is a process, not a one-off event. Defining human death as a “dissociated phenomenon”, although not analyzed in this article, was one of the topics of interviews with doctors conducted by our Interdisciplinary Research Team. The interviews revealed that those who were interviewed presented two opposite references to the definition of human death: namely, some understood death as a fact, and some perceived death as a process. The results of these qualitative studies will be presented in a future article. It is worth noting that just after the research was completed, the Notice of the Minister of Health of 4 December 2019 was introduced, which no longer mentions that “death is a dissociated phenomenon” but that “death is permanent loss of capacity for consciousness and loss of all brain functions”. Such a definition of death in the current law in Poland is not quite consistent with the operational definition of human death (Montreal Forum Report 2012), namely: “Death occurs when there is permanent loss of capacity for consciousness and loss of all brain functions” [following sentence of this Report: “In the context of death determination ‘permanent’ refers to loss of function that cannot resume spontaneously and will not be restored through intervention”] [24]. Although in Polish legislation, brain death is equated with human death determined on the basis of the cardiopulmonary criterion, not all the interviewed physicians chose to accept this statement.

The substantive ambiguity regarding the understanding of human death in the Polish legislation of both 2007 and 2019 inspired our Interdisciplinary Research Team to conduct a survey on the interpretation of human death by early career physicians without specialization and, thus, who are general practitioners. In this work, an assumption was taken that opinions of this pre-specialist physicians are relevant in the medical debate on human death because of the fact that their theoretical formal training is fresh in their mind.

### Aim of the Study

The main aim of the study was to determine the opinions of pre-specialist physicians on their understanding of human death and to discover their beliefs regarding the moment of its occurrence in the light of NMH2007. This is an important problem from both a medical and social point of view, and it has been the subject of scientific debates in the medical community for several decades. It is worth mentioning in this context that a group of specialist physicians was also examined. This article presents the results of research on physicians with a short professional experience, as it enables scientific reflection on the extent to which they know NMH2007 and to what extent they express their own beliefs regarding the understanding of human death. Within the framework of the formulated purpose, the researchers sought answers to the following questions:

1. What is the understanding of human death by early career physicians who do not have specializations in light of NMH2007?
2. Which of the surveyed individuals are in agreement with NMH2007 and which are not, with regard to the condition of the patient’s body that allows for the pronouncement of death after diagnosing irreversible cessation of brain function?
3. According to the respondents, can the irreversible loss of consciousness of the patient constitute a sufficient basis for determining his/her death in relation to the content of NMH2007?
4. What are the areas of compatibility and incompatibility of the respondents’ opinions regarding NMH2017 in relation to cerebral death being synonymous with human death?
5. What is the relationship between selected characteristics of the surveyed group and their understanding of human death according to NMH2007?

Before the start of the research, the authors of this article assumed that all pre-specialist physicians know the definition of human death; they properly understand when a person’s body is dead; they know that the patient’s irreversible loss of consciousness is sufficient grounds for declaring his or her death; they know the criteria for ascertaining human death (cardiopulmonary and neurological), because such knowledge was passed on to them during their medical studies, and, for this reason, it is well preserved in their memory, and this knowledge was in accordance with the legal regulations in force at the time of the research (NMH2007). We also assumed that there would be no differences in their understanding of human death depending on age and seniority. This research was carried out due to the worldwide debate on the border between human life and death and the understanding of the fact of death.

## 2. Materials and Methods

The study was conducted between 1 July 2019 and 30 October 2019, i.e., during the period when NMH2007 was in force. The investigators turned to 200 pre-specialist physicians from various medical centers throughout Poland. These individuals returned 113 completed questionnaires that were then analyzed; hence, the response rate was 56.50%. Those who completed the study were practicing physicians between 25 and 35 years of age, with a mean age of 28.63 ± 1.90 years. Women constituted the majority of the respondents (56.64%). The years of professional experience of the study participants ranged from 1 to 9 years, with a mean value of 2.74 ± 1.52. Most frequently, the physicians worked in teaching hospitals (60.18%), and most did not report deaths as part of their work (69.91%). It should be emphasized that respondents were pre-specialist physicians, and for this reason, due to the legal and medical regulations in force in Poland (NMH2007), they could not confirm the death of patients using cerebral criteria. Detailed data in this regard are presented in Table 2.

The diagnostic survey method was utilized for this study. The research tool consisted of the authors’ test, which included 100 multiple-choice questions (MCQ). The questions were formulated based on the then-binding NMH2007. For the purpose of this article, questions from the authors’ test that directly related to the indicated research issues were used. The content of other test questions will be analyzed in subsequent scientific papers.

Numbers, percentages and descriptive statistics, such as mean, standard deviation, median, minimum and maximum, were employed to describe the results of the study. The Mann–Whitney U test was applied to examine the differences in the age and seniority of the respondents in the context of their opinions on the understanding and timing of human death. A non-parametric test was used due to the lack of normal distribution. The normality of the distribution was tested by means of the Shapiro–Wilk W test. In turn, Pearson’s chi-squared test was utilized to examine the relationship between the respondents’ sex, place of work and frequency of declared deaths, along with their opinions on the understanding and timing of human death.

## 3. Results

The study showed that early career physicians (who have not yet obtained their specializations) understood death in an inconsistent manner. For 43.36% of the respondents, human death was understood as a loss of internal organ integration, i.e., as defined in NMH2007. However, it should be noted that 33.63% of the respondents understood human death more comprehensively, since, in addition to regarding death as the loss of the internal integration of organs, they also took into account that it entails the cessation of being a human, the leaving of the body by the soul and the irreversible loss of consciousness [25,26,27]. It is interesting that for 3.54% of the study participants, death is the end of being a human, while for 19.47%, death means only that the soul leaves the body and that consciousness is irreversibly lost.

Furthermore, the respondents were asked about the vitality of the patient’s body—when a medical board declares death after a diagnosis of irreversible cessation of the brain function has been made. Herein, 7.08% of the surveyed physicians responded in accordance with the provisions of the binding medical and legal act, which indicates that “… the patient’s body is dead when the tissues and organs no longer form an integrated whole, thus they do not constitute a living organism”. In the opinion of the remaining respondents (92.98%), the patient’s body is still alive, although its functionality is limited by the irreversible loss of consciousness (18.59%) or by the fact that the brain is dead (43.36%) or because the brain does not perform activities essential for the organism to coordinate its functioning (30.97%).

The respondents found it problematic to address the question of whether the irreversible loss of consciousness of the patient constitutes sufficient grounds for declaring him or her dead. This question was designed to check to what extent the knowledge of novice physicians regarding death on the basis of irreversible loss of consciousness is consistent with the content of NMH2007. According to 47.79% of the surveyed individuals, none of the answers to the question contained in the test were consistent with the medical and legal act then in force. The remaining 52.21% of respondents based their responses on three concepts found in the literature on the subject, namely: bioethics (loss of personhood) [28]—11.50%; pertaining to religion or spirituality (loss of the soul or the spiritual element of the human person) [29]—11.50%, utilitarianism (predicting the entry into a vegetative state and the inability to return to active life) [30,31]—29.21%.

The research also addressed the legal-medical likening of cerebral death to human death. According to NMH2007, “… the death of a human being as a whole [determined on the basis of cardiopulmonary criterion–authors’ note] in selected cases, is equal to the cerebral death of a human being as a whole”. Our survey indicated that 56.64% of all pre-specialist physicians agreed with this statement. The remaining 43.36% of the respondents provided answers based on current scientific discussion found within world literature on the subject. According to 22.12% of the surveyed physicians, the death of a human being means an irreversible loss of consciousness, for 14.16%, it refers to the loss of the ability to be a human being due to the permanent loss of features characteristic of a human person, and for 7.08%, it denotes the death of the brain treated as the seat of the soul [32,33]. Their opinions are presented in Table 3.

Statistical analysis showed that a majority of the surveyed physicians who were aware of the binding legal and medical act of 2007 did not regard these three types of concepts of human death (bioethical, religious and utilitarian) included in the proposed answers to the MCQ test as consistent with this document. Those who agreed with its content were substantially younger (*p* = 0.001) and worked for a significantly shorter time (*p* = 0.047) than respondents who referred to the above concepts described in the literature. We also found that study participants answering that cerebral death was equal to the biological death of a human being were significantly younger (*p* = 0.002) and had worked for a shorter amount of time as doctors (*p* = 0.009) than did individuals who responded inconsistently with the document. However, there were no significant differences in age and length of service among respondents whose answers were based on the content of the document and those who chose answers that had no reference to this content, both in terms of the understanding of death and the condition of the patient’s body allowing the determination of death after diagnosing the irreversible cessation of the brain function. The said significance is shown in Table 4.

The responses received indicate that women were significantly more likely (*p* = 0.028) to believe that death should be defined based on the legal and medical document applicable in NMH2007 and, in accordance with this, significantly more often (*p* = 0.040) did not choose answers that were based on the three aforementioned types of concepts of human death. They also did not indicate on what basis cerebral death is equivalent to human death (*p* = 0.003). In contrast, no significant association was found between the place of work, the determination of deaths and the pre-specialist physicians’ opinions on the understanding of death. See Table 5.

Questions aimed at identifying the opinions of pre-specialist physicians on the moment of human death were important from the point of view of the long-standing scientific discussion on this matter. The research showed that 42.48% of the surveyed individuals indicated the moment when human death occurs according to NMH2007. The remaining 57.52% of respondents provided incomplete answers or answers inconsistent with the content of this document. The data on this matter are presented in Table 6.

The statistical analysis revealed no significant differences in age and seniority depending on the pre-specialist physician (who have been out of medical school for 9 years or less) opinions on the timing of human death. See Table 7.

No significant relationship was found between sex, place of work, as well as determination of death and pre-specialist physicians’ opinions on the timing of human death. However, it can be noted that physicians who declared death at their workplace were slightly more likely to agree with the content of the 2007 legal and medical document (32%, as compared to respondents who did not declare death (19%). See Table 8.

## 4. Discussion

Researchers expected confirmation of the hypotheses. Indeed, we assumed that all pre-specialist physicians knew the definition of human death as provided in the then applicable Polish legislation (NMH2007). This assumption was based on the fact that they had recently graduated from medical school, during which they had to acquire knowledge about human death and its determination. The conducted research did not confirm the assumed hypothesis, although physicians familiar with the content of the legal and medical document (NMH2007) had significantly less seniority. We also assumed that all respondents agreed with the statement in NMH2007 that “death has already reached the brain, while the blood circulation is still preserved”—that is, the body is alive. This hypothesis was not confirmed because the answers chosen by the respondents were very diverse. This may indicate either ignorance of NMH2007 or disagreement with its content. Another hypothesis was the assumption that all subjects knew that in NMH2007, the irreversible loss of consciousness of the patient does not constitutes sufficient grounds for declaring him or her dead, but “the irreversible cessation of the brain-stem function” does. This hypothesis was also not confirmed, as the analysis of the research results shows that 47.79% of the respondents chose the answer consistent with NMH2007, which was in force at the time. It is worth noting that the death of a human being is not unambiguously understood by physicians without specialization, as their response choices were based on anthropological and bioethical as well as religious or medical beliefs. The last hypothesis was the assumption that the respondents’ understanding of death had no significant relationship with their age and seniority. However, the research showed a statistically significant relationship between the knowledge of NMH2007 and their age and seniority. Younger physicians and physicians with shorter seniority more often chose answers in accordance with applicable law (NMH2007). Of note: the Interdisciplinary Research Team, in addition to this research, has conducted research among specialist physicians on the subject of understanding human death. Currently, the results of these studies are being developed and prepared for publication. The authors also plan to conduct research within other social groups, especially lawyers.

The world’s first heart transplantation took place on 3 December 1967. It was performed by Dr Christiaan Barnard at the Groote Schuur Hospital in Cape Town, South Africa. This event sparked a worldwide scientific discussion and a media debate on issues concerning both the boundary between human life and death and the understanding of death itself. The debate included questions, such as whether heart donors are dead if their hearts are still alive. Issues had also been raised about the scientific basis for the legal authorization of organ donation procedures and whether cerebral death can be compared to death established on the basis of cardiopulmonary criteria [34].

In the scientific debate on brain death, the following are pointed out: incoherence between concept and criterion, incoherence between criterion and tests, incoherence between tests and concept, and incoherence between theory and practice [35]. Various types of inconsistencies in concept, criteria, practice and documentation of brain death/death by neurological criteria were the reason why relevant international professional societies developed recommendations on determination of brain death/death by neurological criteria. This World Brain Death Project has had as its objective the formulation of a consensus statement of recommendations on determination of brain death/death by neurological criteria, taking into account both review of the literature and expert opinions [36].

In Poland, between 2007 and 2019, the basic legal act providing grounds for declaring the cerebral death of a patient was NMH2007 (Dz. U./Journal of Laws/No. 169 item 1411). This document specified the criteria and method of establishing the permanent and irreversible cessation of brain function, which were determined by specialists in four branches of medicine, namely: anesthesiology and intensive care, neurology, neurosurgery and forensic medicine. According to NMH2007, it is “the condition of the brain [that] determines the life or death of a person”. In reference to the neurological criteria applicable in many countries of the world, the Polish legislation also specified that “the qualifying factor of cerebral death is the irreversible absence of the brainstem function”. The authors of the General Assumptions of NMH2007, who were professors of medical sciences, were aware that the clinical symptoms of brainstem damage do not necessarily always indicate “simultaneous irreversible damage to the entire brain”. Therefore, they stated that it is necessary to confirm this diagnosis using instrumental tests. Referring to the thesis that “many years of medical practice have unequivocally shown that in selected cases, the abandonment of the concept of death of a human being as a whole in favor of the death of a human brain as a whole is justified from a scientific and practical point of view”, they believed that “such a position proves to be necessary and right by all means”.

In Poland, due to the need to adapt to global legal and medical achievements in the understanding of human death and cerebral death, as well as to clarify the neurological criteria for its determination, a new document was prepared by a team of medical specialists with regard to the understanding of human death, namely, NMH2019 (Dz. U./Journal of Laws/of 2019, item 537, 577, 730 and 1590). Compared to the legal document on the same topic issued in 2007, the current criteria and procedure for determining the permanent and irreversible cessation of the brain function have been clarified by a team of specialists from fifteen medical disciplines. The document begins with the observation that “[t]he criteria for human death evolve along with the progress of medical knowledge and are periodically revised by consensual agreements leading to unanimity by the scientific medical community”. In terms of the progress of medical knowledge, it is important to note that until the second half of the twentieth century, despite the centuries-long development of knowledge about the diagnosis and treatment of human diseases and various discoveries within medical science, the criterion of human death did not undergo any changes. The respiratory and circulatory criterion was perceived as certain and sufficient to determine whether a person was alive or dead. The dependence of the criteria of death on “agreements leading to unanimity”, which resulted from debates within the scientific medical community, can hardly be regarded in empirical sciences as constituting an unquestionable basis. Therefore, it should not come as a surprise that a number of questions have been raised in professional medical debate and on the Internet about whether the reliance on the social agreement of medical specialists with regard to the criteria for determining death could lead to relativistic and pragmatic attitudes in pursuit of goals, such as, for example, the greater availability of organs from “dead donors” for transplants. It is worth noting that the currently applicable NMH2019 states that “[t]he evolution of the criteria of human death in each case, however, confirms that the case-law to date has been and remains reliable and confirms the biological fact of death”. The second paragraph of this document quotes a slightly modified definition of death, which was initially formulated in 2012 during an international expert meeting in Montreal [24]. The General Assumptions of Appendix 1 to the aforementioned NMH2019 define that “[d]eath is a permanent loss of consciousness and a permanent loss of all brainstem functions.” This may be caused by the permanent cessation of circulation or by critical brain damage. In the context of determining death, the term “permanent” implies a loss of function that cannot return spontaneously nor be restored through intervention. However, it is important to note that “the Operational Definition of Human Death” from the Montreal Forum Report does not contain a precise definition of human death, only a statement that death occurs. Taking into account the existing debate on the understanding of human death and cerebral death, the medical experts gathered in Montreal probably took it for granted that death is essentially a mystery and thus cannot be precisely defined. After all, death is more part of the ontological rather than the biological order. At the biological level, it is only possible to observe changes that take place in the body when they occur. For this reason, the Montreal Forum Report does not define the notion of death but rather indicates that death “occurs” or “happens” when certain conscious-organic changes take place. It is perplexing why the phrase “death is” was used in NMH2019. According to the Polish legislator, “death is a permanent loss of capacity for consciousness and a permanent loss of all brainstem functions” ascertained through instrumental tests. This type of definition of human death raises a number of questions, including those of an interdisciplinary nature. While from a biological perspective, it is reasonable to assume that human death occurs as a result of the permanent loss of all brainstem functions, it is questionable whether the current diagnostic methods provide 100% certainty that a human being in that state is definitely dead. Why should the maintenance of consciousness constitute a confirmation that a person is alive and a definite loss of consciousness suggests the possibility that a person is dead? On what grounds, in relation to humans, is it reasonable to associate communicable consciousness with life and incommunicable consciousness with death?

It is worth noting that up to this point, no uniform international criteria for determining cerebral death have been formed. Over the last few decades, the neurological criteria for death have been constantly refined to provide as much certainty as possible that brain activity has completely ceased. However, the neurological criteria of human death raise various types of doubts, particularly in relation to anthropological concepts representative of various religions of the world. Despite many discussions on the understanding of human death, including its very moment, as well as cerebral death and the neurological criteria necessary for its determination, this issue has yet to be studied to a sufficient extent in relation to the opinions of both early career physicians without specialization and specialists. Studies on the understanding of cerebral death and the criteria for its establishment by physicians have been conducted since the 1980s. Indeed, a cross-sectional survey of knowledge and concepts of brain death among health professionals was conducted in the USA in 1989 [37], while a survey of American neurologists about brain death was carried out in 2012 to investigate the conceptual basis of and diagnostic tests for cerebral death (The research clearly shows that American neurologists do not have a consistent rationale for accepting brain death as death, or a clear understanding of diagnostic tests for brain death.) [38]. Moreover, a 2006 survey of neurosurgeons (the survey results indicate that there is a tremendous disparity between Canadian neurosurgeons’ opinions on the understanding of the conceptual basis and diagnostic tests used for brain death. The equivalence of cerebral death with death established on the basis of cardiopulmonary criteria was ambiguously understood by the respondents. It is particularly puzzling that 39% of all neurosurgeons chose a prognosis concept of death) [39] and pediatricians working in intensive care units [40] conducted in Canada proved to be highly controversial among the medical community. Furthermore, studies conducted in the Baltic states [41], Italy (2001) [42], Turkey (2003) [43], Korea (2004) [44], Brazil (2005) [45], Pakistan (2008) [46] and Brazil (2016) [47] significantly contributed to the discussion on cerebral death and organ donation. Indeed, in 2017, surveys on the subject of the awareness of brain-death declaration needed for cadaver organ donation were conducted in India among resident physicians [48]. Their results revealed that over 90% of all resident physicians knew the criteria for determining brain death that were in force in India at that time, which is in accordance with applicable law.

Although according to the currently applicable Polish law, human death based on a set of neurological criteria can only be determined by a medical board consisting of two specialists (a neurologist and an anesthesiologist), the opinions of early career physicians without specialization can be very helpful in analyzing the issue of the understanding of death established on the basis of both the cardio-respiratory criterion and cerebral death in relation to broadly understood public opinion.

Contradictory to the hypothesis put forward by the researchers, the study showed (despite the surveyed physicians being a homogeneous group—young in seniority and age, without specializations) an inconsistency between the understanding of death and the legislation applicable in Poland—which specifies this understanding of human death and cerebral death. The differences were statistically significant. A questioning of whether the understanding of human death and cerebral death by pre-specialist physicians was conditioned by their unawareness of the then binding NMH2007, or their disagreement with the theses contained in that document, and to what extent such understanding resulted from their beliefs based on the literature on the subject, which also presented other concepts of human death (particularly the bioethical, religious or utilitarian concepts), appears to be obvious. Why was such a small percentage of pre-specialist Polish physicians in agreement with the binding document as to when a human body can be considered dead? This also raises the question of what conditioned their belief that the irreversible loss of consciousness does not constitute sufficient grounds for declaring a person dead. However, it is worth considering that physicians with shorter seniority were significantly more inclined to agree with the content of the applicable legal and medical act than those with longer seniority. The research revealed a surprising fact that as many as 57.52% of all respondents provided incomplete answers or answers that did not comply with the content of the NMH2007 pertaining to the question about the timing of human death.

The studies that we conducted on a group of pre-specialist Polish physicians not only encourage reflection on their results but also clearly indicate the need to learn more about the understanding of human death by medical specialists—particularly by anesthesiologists, neurologists, neurosurgeons, transplantologists and cardiologists—in relation to the relevant law currently in force in Poland.

## 5. Conclusions

1. Early career physicians without specialization had an ambiguous understanding of human death. The vast majority of respondents perceived human death in a manner inconsistent with the binding NMH2007.
2. According to a small percentage of the surveyed individuals, the patient’s body is dead when the tissues and organs no longer form an integrated whole and therefore do not constitute a living organism. This type of opinion is consistent with the above-mentioned legal and medical document.
3. A smaller percentage of the study participants agreed with the content of the binding medical and legal act regarding the irreversible loss of a patient’s consciousness as sufficient grounds for declaring him or her dead, whereas the majority of respondents believed that the concepts of human death (bioethical, religious and utilitarian) found in the literature on the subject were valid.
4. For most of the studied subjects, human death was determined on the basis of cardiopulmonary criteria in accordance with the applicable medical procedures as well as with the criteria for determining death included in NMH2007 (death of the human brain as a whole).
5. The statistical analysis showed significant differences between age and seniority in the context of responses regarding the irreversible loss of consciousness of the patient as a sufficient basis for determining his or her death, while NMH2007 sees cerebral death as equal to biological death.

## Figures and Tables

**Table 1:** Differences in Polish legislation (NMH2007 and NMH2019) in defining human death and the criteria for its determination.

Legal Act	The Definition of Human Death	The Criterion that Determines a Human’s Life or Death	Brain Death Criteria	Criteria for Brain Stem Damage
NMH2007	“Death is a dissociated phenomenon.”	“The state of the brain determines a person’s life or death.”	“The qualifying factor for brain death is the irreversible lack of brainstem function.”	“Permanent damage to the brainstem is determined by specific nervous reflexes and the absence of spontaneous breathing.”
NMH2019	“Death is the permanent loss of consciousness and the permanent loss of all functions of the brainstem.”	Undefined.	“Critical brain injury leads to a diagnosis of death based on neurological criteria, traditionally referred to as brain death.”	“In most cases, the brain edema resulting from its damage increases from the side of the supratentorial space, and the brain stem dies as the last part of it. Permanent damage to the brainstem is determined by the absence of specific nervous reflexes and the absence of spontaneous breathing.”

**Table 2:** Characteristics of the study group.

Variables		M ± SD	Me (Min; Max)
Age (years)		28.63 ± 1.90	28 (25; 35)
Seniority (years)		2.74 ± 1.52	3 (1; 9)
		N	%
Sex	Female	64	56.64
	Male	49	43.36
Place of work	Teaching hospital	68	60.18
	Regional hospital	30	26.55
	City/district hospital	15	13.27
Frequency of declared deaths	I declare no deaths	79	69.91
	Several times a year	20	17.70
	Several times a month	11	9.73
	More than several times a month	3	2.65

**Table 3:** Opinions of pre-specialist physicians on the understanding of human death.

Statement		*N*	%
I understand human death as:	the cessation of being a human person.	4	3.54
	the leaving of the body by the soul and the irreversible loss of consciousness.	22	19.47
	the loss of the internal integration of organs.	49	43.36
	something that requires all the above criteria to be met.	38	33.63
When a medical board declares death after diagnosing the irreversible cessation of the brain function, the patient’s body is:	alive, but he or she has lost consciousness irreversibly.	21	18.59
	alive, but his or her brain is dead.	49	43.36
	alive, but his or her brain does not perform the essential functions that coordinate the functioning of the body.	35	30.97
	dead, as there has been a disintegration of the organism as a functional whole caused by the collapse of its individual functions over time.	8	7.08
The irreversible loss of consciousness of the patient constitutes sufficient grounds for declaring him or her dead since it means that:	the patient has lost the characteristics proper to a human person (loss of personhood).	13	11.50
	the patient’s soul or spiritual element has left his or her body, making it impossible for him or her to be a human being.	13	11.50
	the patient is likely to enter a vegetative state and be unable to return to active life.	33	29.21
	the above answers are not contained in the current *NMH2007*.	54	47.79
Brain death is associated with the death of the human being because:	it represents an irreversible loss of consciousness.	25	22.12
	it denotes the death of the brain, regarded as the seat of the soul.	8	7.08
	it entails the loss of the capacity to be a human being due to the permanent loss of the characteristics specific to the human person.	16	14.16
	it is synonymous with the biological death of a human being.	64	56.64

**Table 4:** Age and length of service of pre-specialist physicians in the context of their opinion on the understanding of human death.

Statement	Reference	Age (Years)			Mann–Whitney U test		Seniority			Mann–Whitney U Test	
		Me	M	SD	Z	*p*	Me	M	SD	Z	*p*
Understanding of death in relation to *NMH2007.*	Consistent (*n* = 38)	29	28.84	1.97	0.978	0.328	3	3.03	1.67	1.422	0.155
	Inconsistent (*n* = 75)	28	28.52	1.88			2	2.60	1.43		
Pre-specialist physicians’ belief in the guidelines contained in *NMH2007* concerning the condition of the patient’s body after the confirmation of death due to the irreversible cessation of brain function.	Consistent (*n* = 8)	29.5	29.50	3.66	0.705	0.481	3	3.75	2.66	1.069	0.285
	Inconsistent (*n* = 105)	28	28.56	1.71			2	2.67	1.39		
Pre-specialist physicians’ belief that the irreversible loss of consciousness of a patient constitutes sufficient grounds for declaring him or her dead according to *NMH2007.*	Consistent (*n* = 54)	28	28.09	1.90	−3.225	0.001	2	2.50	1.53	−1.986	0.047
	Inconsistent (*n* = 59)	29	29.12	1.79			3	2.97	1.50		
Pre-specialist physicians’ belief in the stipulation contained in the *NMH2007* that cerebral death is synonymous with human death.	Consistent (*n* = 64)	28	28.16	1.78	−3.085	0.002	2	2.47	1.48	−2.610	0.009
	Inconsistent (*n* = 49)	29	29.24	1.90			3	3.10	1.52		

**Table 5:** Opinions of pre-specialist physicians on the understanding of human death depending on sex, place of work and frequency of declared deaths.

Statement	Reference	Sex		Pearson’s Chi-Squared Test		Place of Work			Pearson’s Chi-Squared Test		Declaration of Deaths		Pearson’s Chi-Squared Test	
		F	M	Chi^2	*p*	TH	RH	CH/DH	Chi^2	*p*	Yes	No	Chi^2	*p*
Understanding of death in relation to *NMH2007.*	Consistent (*n* = 38)	27 (42%)	11 (22%)	4.844	0.028	27 (40%)	5 (17%)	6 (40%)	5.265	0.072	14 (41%)	24 (30%)	1.241	0.265
	Inconsistent (*n* = 75)	37 (58%)	38 (78%)			41 (60%)	25 (83%)	9 (60%)			20 (59%)	55 (70%)		
Pre-specialist physicians’ belief that the irreversible loss of consciousness of a patient constitutes sufficient grounds for declaring him or her dead according to *NMH2007.*	Consistent (*n* = 54)	36 (56%)	18 (37%)	4.236	0.040	30 (44%)	18 (60%)	6 (40%)	2.525	0.283	14 (41%)	39 (49%)	0.263	0.608
	Inconsistent (*n* = 59)	28 (44%)	31 (63%)			38 (56%)	12 (40%)	9 (60%)			20 (59%)	40 (51%)		
Pre-specialist physicians’ belief in the stipulation contained in *NMH2007* that cerebral death is synonymous with human death.	Consistent (*n* = 64)	44 (69%)	20 (41%)	8.817	0.003	39 (57%)	19 (63%)	6 (40%)	2.252	0.324	15 (44%)	39 (49%)	0.271	0.603
	Inconsistent (*n* = 49)	20 (31%)	29 (59%)			29 (43%)	11 (37%)	9 (60%)			19 (56%)	40 (51%)		

Legend: F—female; M—male; TH—teaching hospital; RH—regional hospital; CH/DH—city hospital/district hospital.

**Table 6:** The moment of human death according to pre-specialist physician opinion.

Statement		*N*	%
Human death occurs:	at the moment of cardiac arrest.	19	16.81
	20 min after cardiac arrest at room temperature.	26	23.01
	at the moment when the medical board signs a document declaring the cerebral death of the patient.	20	17.70
	20 min after cardiac arrest at room temperature or at the moment when the medical board signs a document declaring the cerebral death of the patient.	48	42.48

**Table 7:** Age and seniority of pre-specialist physicians depending on their opinion on the timing of human death.

Question	Answer	Age (Years)			Mann-Whitney U Test		Seniority (Years)			Mann-Whitney U Test	
		Me	M	SD	Z	*p*	Me	M	SD	Z	*p*
When does human death occur?	Consistent with *NMH2007* (*n =* 26)	29	28.65	1.55	−0.515	0.607	3	2.69	0.93	−0.515	0.607
	Inconsistent with *NMH2007* (*n* = 87)	28	28.62	2.01			2	2.76	1.66		

**Table 8:** Opinions of pre-specialist physicians on the timing of human death depending on sex, place of work and frequency of declared deaths.

Question	Answer	Sex		Pearson’s Chi-Squared Test		Place of Work			Pearson’s Chi-Squared Test		Declaration of Deaths		Pearson’s Chi-Squared Test	
		F	M	Chi^2	*p*	TH	RH	CH/DH	Chi^2	*p*	Yes	No	Chi^2	*p*
When does human death occur?	Consistent with *NMH2007* (*n* = 26)	17 (27%)	9 (18%)	1.052	0.305	15 (22%)	9 (30%)	2 (13%)	1.655	0.437	11 (32%)	15 (19%)	2.397	0.122
	Inconsistent with *NMH2007* (*n* = 87)	47 (73%)	40 (82%)			53 (78%)	21 (70%)	13 (87%)			23 (68%)	64 (81%)		

Legend: F—female; M—male; TH—teaching hospital; RH—regional hospital; CH/DH—city hospital/district hospital.

## Data Availability

The data presented in this study are available on request from the corresponding author.
